# HDAC inhibitors as cognitive enhancers in fear, anxiety and trauma therapy: where do we stand?

**DOI:** 10.1042/BST20130233

**Published:** 2014-03-20

**Authors:** Nigel Whittle, Nicolas Singewald

**Affiliations:** *Department of Pharmacology and Toxicology, Institute of Pharmacy and Center for Molecular Biosciences Innsbruck (CMBI), University of Innsbruck, Innrain 80-82/III, A-6020 Innsbruck, Austria

**Keywords:** anxiolytic therapy, cognitive enhancer, epigenetics, histone acetyltransferase activator (HAT activator), histone deacetylase inhibitor (HDAC inhibitor), lysine acetylation, trauma, BDNF, brain-derived neurotrophic factor, CBP, CREB-binding protein, CBT, cognitive behavioural therapy, CoREST, co-repressor for element-1-silencing transcription factor, CREB, cAMP-response-element-binding protein, CS, conditioned stimulus, HAT, histone acetyltransferase, HDAC, histone deacetylase, NMDA, *N*-methyl-D-aspartate, NuRD, nucleosome remodelling and deacetylation, PTSD, post-traumatic stress disorder, SAHA, suberoylanilide hydroxamic acid, SPS, single prolonged stress, SSRI, selective serotonin-re-uptake inhibitor, US, unconditioned stimulus, VPA, valproic acid

## Abstract

A novel strategy to treat anxiety and fear-related disorders such as phobias, panic and PTSD (post-traumatic stress disorder) is combining CBT (cognitive behavioural therapy), including extinction-based exposure therapy, with cognitive enhancers. By targeting and boosting mechanisms underlying learning, drug development in this field aims at designing CBT-augmenting compounds that help to overcome extinction learning deficits, promote long-term fear inhibition and thus support relapse prevention. Progress in revealing the role of epigenetic regulation of specific genes associated with extinction memory generation has opened new avenues in this direction. The present review examines recent evidence from pre-clinical studies showing that increasing histone acetylation, either via genetic or pharmacological inhibition of HDACs (histone deacetylases) by e.g. vorinostat/SAHA (suberoylanilide hydroxamic acid), entinostat/MS-275, sodium butyrate, TSA (trichostatin A) or VPA (valproic acid), or by targeting HATs (histone acetyltransferases), augments fear extinction and, importantly, generates a long-term extinction memory that can protect from return of fear phenomena. The molecular mechanisms and pathways involved including BDNF (brain-derived neurotrophic factor) and NMDA (*N*-methyl-D-aspartate) receptor signalling are just beginning to be revealed. First studies in healthy humans are in support of extinction-facilitating effects of HDAC inhibitors. Very recent evidence that HDAC inhibitors can rescue deficits in extinction-memory-impaired rodents indicates a potential clinical utility of this approach also for exposure therapy-resistant patients. Important future work includes investigation of the long-term safety aspects of HDAC inhibitor treatment, as well as design of isotype(s)-specific inhibitors. Taken together, HDAC inhibitors display promising potential as pharmacological adjuncts to augment the efficacy of exposure-based approaches in anxiety and trauma therapy.

## Introduction

Anxiety and fear-related disorders, including phobias, generalized anxiety disorder, panic disorder, post-traumatic stress disorder and obsessive compulsive disorder, are disabling, persistent and extremely prevalent disorders as epidemiology surveys estimate that up to 30% of the Western world populations will experience one or more of these disorders during their lifetime [1–3]. Current treatments of anxiety disorders involve pharmacotherapy [e.g. antidepressants such as SSRIs (selective serotonin-re-uptake inhibitors) and anxiolytics such as benzodiazepines and gabapentin] and/or exposure-based CBT (cognitive behavioural therapy). However, and aside from the well-described side effects of chronic anxiolytic pharmacotherapy, these treatments are not always successful as a significant proportion of patients remain symptomatic after their initial intervention [4,5] and return of fear is commonly reported [6]. Attempts to enhance treatment response by combining CBT and anxiolytic pharmacotherapy has so far not yielded a convincing beneficial effect in fear reduction compared with either CBT or anxiolytic treatment alone [4,7]. Thus there is a clear need to develop novel strategies to elicit more effective interventions that promote enduring fear inhibition with reduced side effects in anxiety disorder patients.

Recently, a novel strategy has emerged to treat anxiety disorder patients by combining cognitive enhancers (i.e. substances that can enhance mental functions such as cognition, memory, intelligence and concentration in humans) with CBT. This strategy has been driven primarily by the translation of pre-clinical research identifying brain circuitry and molecular underpinnings of exposure-based fear extinction/CBT into clinical trials [8]. These studies have revealed that NMDA (*N*-methyl-D-aspartate) receptor- mediated signalling is one of the critical molecular mechanisms underlying exposure-based fear extinction [9–11] and suggest that enhancing NMDA receptor activity during CBT may act as a cognitive enhancer strategy to augment fear reductions. Indeed, as a prime example of successful translational research, clinical studies have revealed that enhancing NMDA receptor activity, via administration of the NMDA receptor partial agonist D-cycloserine, augments fear extinction induced by CBT in subjects with anxiety disorders such as acrophobia, height phobia, social anxiety disorder, obsessive compulsive disorder and panic disorder [11–14]. The drug seems to work primarily by consolidating extinction memory gained during successful exposure training sessions; however, D-cycloserine can actually impair fear extinction if no significant reduction in fear during extinction/CBT training is achieved [13,15–17]. D-Cycloserine may not always exert optimal efficacy, as there is evidence that, for example in social anxiety patients, the achieved fear inhibition may not be sustained [14].

The quest to reveal additional novel cognitive enhancers to augment CBT and promote long-term fear inhibition is ongoing [18,19] and translational research has targeted, for example, the endocannabinoid [20] or dopaminergic [21] systems. On the basis of advances in the knowledge of the role of epigenetic mechanisms in the regulation of genes critical for memory formation (for a recent review, see [22]), results reveal that enhancing gene transcription, via increasing histone acetylation, can enhance fear extinction memories (described in greater detail below). This approach seems particularly attractive to pursue as results from learning and memory experiments have demonstrated that enhancing specific gene transcription generates a form of long-term memory that is persistent and lasts beyond the duration of normal memory [23,24]. The present review examines existing evidence from pre-clinical studies using rodent models of fear extinction and small clinical studies showing that drugs that increase histone acetylation can indeed augment fear extinction and promote sustained fear inhibition. The summarized findings highlight the possibilities of using such pharmaceutical compounds as cognitive enhancers to be combined with psychotherapeutic approaches for the treatment of anxiety disorders.

## Quantifying fear and fear extinction in pre-clinical animal models

Pavlovian fear conditioning has been used extensively to reveal brain circuitry and molecular mechanisms leading to the formation and extinction of fear learning. After repeated pairing of a neutral non-fearful CS (conditioned stimulus) (usually a tone or light) with a US (unconditioned stimulus) (usually a footshock), the CS comes to elicit conditioned fear responses such as freezing, increased startle reflexes, autonomic changes, analgesia or behavioural response suppression [25]. Conditioned fear responses can be attenuated (‘extinguished’) by repeatedly presenting the CS without the US during an extinction training session. Thus fear extinction has been considered to reflect a form of exposure-based CBT [26]. This form of extinction generally does not directly modify the existing fear memory, but rather leads to the formation of a new inhibitory memory that suppresses expression of the initial fear memory [27]. Thus the re-emergence of a previously extinguished fear has been shown to occur in rodents by: (i) renewal, when the CS is presented outside of the extinction context; (ii) reinstatement, when stressors such as the original US are given unexpectedly; or (iii) spontaneous recovery, when a significant period of time has passed [27]. Similarly, also in clinical settings, re-emergence of fear has been observed due to spontaneous recovery [28–30], reinstatement [28] or renewal [31]. Stronger and enduring extinction memory generated in exposure therapy is therefore an important aim to better protect from return of fear phenomena. However, there is evidence that patients with anxiety disorders and PTSD show impaired extinction which limits the effect of exposure-based therapies (for a review, see [32]). Recent research has identified a number of rodent models demonstrating impaired fear extinction (reviewed in [32]) which can mimic patients with resistance to exposure therapy. Using such models, it has been shown that D-cycloserine is ineffective when no extinction training effect is achieved [17], but can rescue deficits in extinction memory consolidation after fear reduction has been induced during extinction training [16].

## HDAC inhibitors as an approach to augment extinction/CBT effects: evidence from animal and human studies

### Relationship of histone acetylation to extinction memory

**Figure 1:**
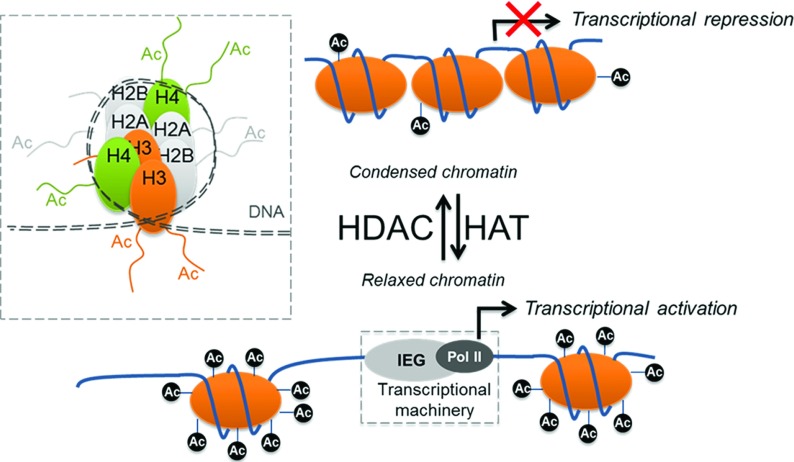
Histone acetylation: a mechanism regulating gene expression Acetylation of histone proteins is catalysed by the action of HATs and is reversed by the action of HDACs. Acetylation can promote gene transcription by (among other mechanisms) causing direct structural changes to chromatin to result in a more relaxed state. This relaxed chromatin state can expose strands of DNA to transcriptional machinery, which is composed of immediate-early genes (IEG, e.g. Zif268, c-Fos) and DNA polymerases (e.g. Pol II), which then can initiate gene transcription. Inset: the core unit of chromatin is the nucleosome. This is an octamer of two molecules of each core histone H2A, H2B, H3 and H4, wrapped around 147 bp of DNA [36]. Ac, acetylated lysine residues on histone tail proteins.

**Figure 2:**
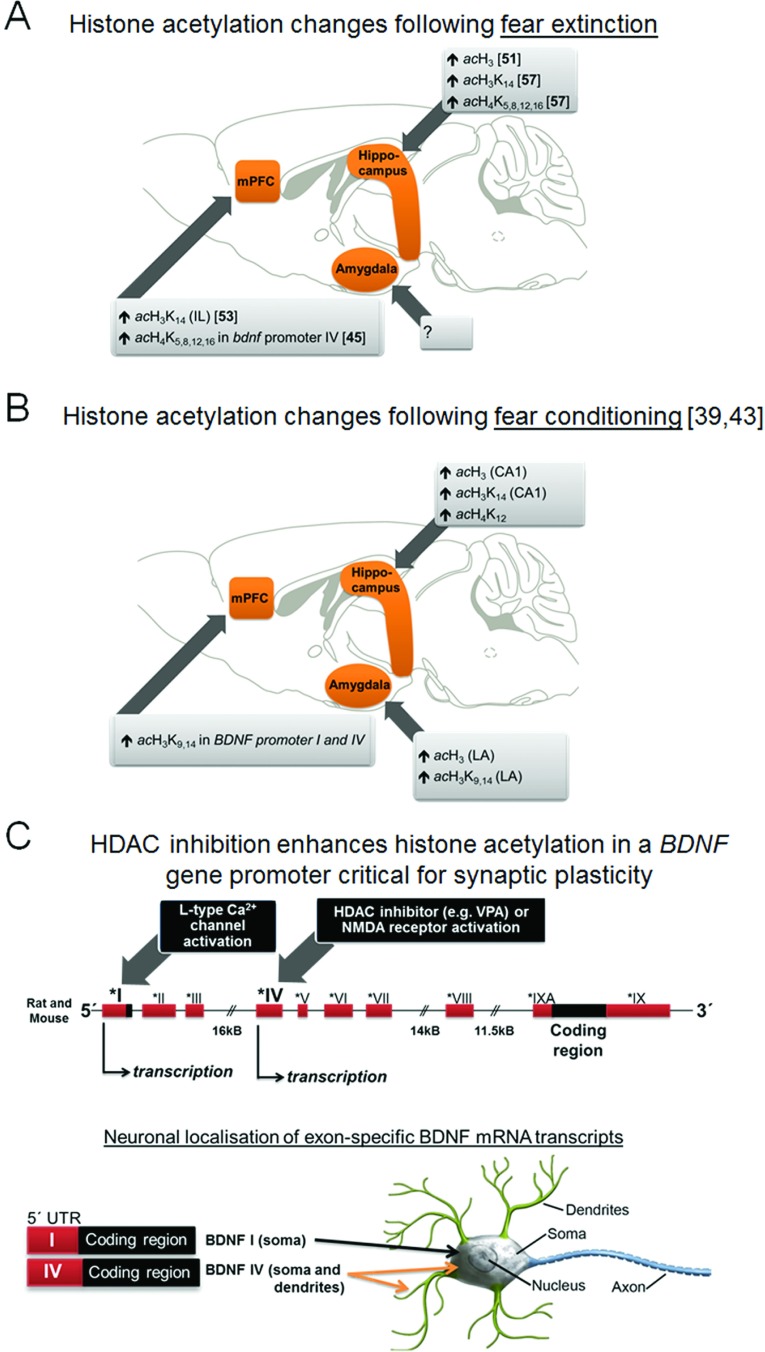
Brain regions displaying enhanced histone acetylation following fear extinction and fear learning Published studies have revealed that successful fear extinction (**A**) and fear conditioning (**B**) is associated with increases in histone H3 and H4 acetylation in the medial prefrontal cortex (mPFC), hippocampus and amygdala (fear conditioning only; there is no published study concerning fear extinction). Differential epigenetic regulation of *BDNF* is observed between fear extinction and fear conditioning in the mPFC: extinction-induced increases in histone H4 acetylation are in the promoter region IV of *BDNF*, whereas fear conditioning increases histone H3 acetylation in promoters of *BDNF*. This differential histone acetylation in *BDNF* promoters between fear extinction and fear conditioning may be of significance as acetylated (*ac*) H3 and H4 are thought to subserve different functions [44]. The rodent *Bdnf* gene contains at least nine 5′ non-coding exons, each with its own promoter, and a common coding exon (**C**). Transcription of *BDNF* transcripts containing exon I or IV has been shown to respond differentially to divergent stimuli; the pan-HDAC inhibitor VPA [82] or NMDA receptor activation predominately increases exon IV-specific mRNA transcripts, whereas L-type voltage-dependent Ca^2+^ signals seem to mostly increase exon I-specific mRNA transcripts [102]. The neuronal localization of exon I-containing *BDNF* transcripts is predominately in the soma, whereas that of exon IV-containing *BDNF* transcripts is in proximal dendrites and in the soma [103]. The targeting of mRNA to specific subcellular compartments, particularly in dendrites is an important feature linked to synaptic plasticity. Thus fear extinction-induced increases in *BDNF* exon IV transcripts in dendrites [which may be potentiated with HDAC inhibitors (Box 1)] may be a significant mechanism underlying successful fear extinction.

**Figure 3:**
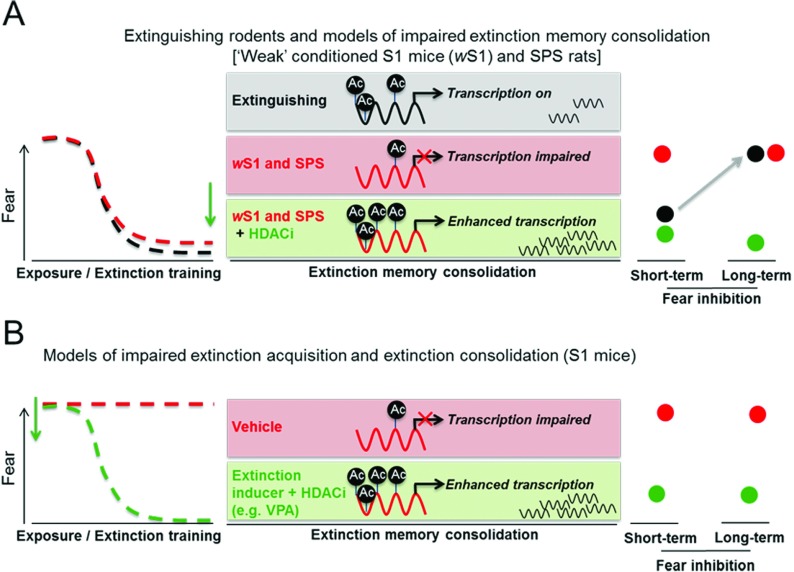
HDAC inhibitors augment fear extinction and rescue cognitive deficits in animal models of impaired extinction (**A**) Normal extinguishing rodents display fear reduction during a exposure-based fear extinction training session (termed ‘extinction acquisition’; black broken line) and extinction-induced increases in histone acetylation (black filled circle) during extinction memory consolidation. Evidence of successful fear extinction consolidation is observed with low fear levels during a retrieval test 24 h later (short-term extinction retrieval; black filled circle). However, long-term fear inhibition is lacking as return of fear can be observed. Weak conditioned S1 (*w*S1) mice and SPS rats display intact extinction acquisition (red broken line), but are unable to consolidate/retrieve this memory (red filled circle), probably due to insufficient increases in extinction-induced histone acetylation and subsequent gene expression changes. Post-extinction training administration of HDAC inhibitors (MS-275, SAHA; green arrow) in *w*S1 and SPS rats can induce histone acetylation with subsequent rescue of extinction-consolidation deficits and promotion of long-term fear inhibition (green filled circles). (**B**) Normal conditioned S1 display in addition also impaired extinction acquisition (red broken line). Pharmaceutical adjuncts such as VPA (green arrow) that induce extinction acquisition (positive training effect, i.e. fear reduction), as well as HDAC inhibition, are required to rescue the pronounced extinction deficits and promote long-term fear inhibition (green filled circles) in these mice. Abbreviations: Ac, acetylated histone; HDACi, HDAC inhibitors.

The consolidation of long-term fear extinction memories requires protein synthesis, a process which involves the co-ordinated transcription of specific genes coding for learning-associated transcription factors, neurotransmitter receptors, cytoskeletal proteins and other cellular substrates [33,34]. An initiating first step requires enzymes that modify chromatin structure to allow transcriptional machinery access to the promoter region of these genes [35]. Chromatin structures consist of strands of DNA (146 bp) wrapped around an octamer of four histone proteins (H2A, H2B, H3 and H4) [36] (Figure 1). Histone modifications have attracted much attention because of their central role in gene regulation. The N-terminal tails of the histones can be modified by acetylation, methylation, phosphorylation, ubiquitination and SUMOylation [37], and these post-translational modifications influence gene transcription. As the present review is focused on histone acetylation, only the mechanisms of how histone acetylation affects gene transcription are described (for a recent review of all known histone post-translational modifications, see [38]). Acetylation and deacetylation of lysine residues on histone tails is regulated by the opposing action of HATs (histone acetyltransferases), also known as KATs (lysine acetyltransferases) and termed ‘writers’, and HDACs (histone deacetylases), termed ‘erasers’ (Figure 1). Histone acetylation is associated with active gene transcription, whereas reduced or no histone acetylation represses gene transcription [35]. Although highly dynamic, histone acetylation via promotion of relevant gene transcription is believed to produce long-lasting changes that sustain physiological processes and behaviour [39]. To date, 18 different mammalian HDAC isoforms have been identified and divided into four classes (classes I–IV), of which class I HDACs comprise HDAC1, HDAC2, HDAC3 and HDAC8 isoforms. Of these HDACs, HDAC1 and HDAC2 are the most extensively studied. HDAC1 and HDAC2 are found in distinct multiprotein complexes either together [Sin3, NuRD (nucleosome remodelling and deacetylation) and NCoR (nuclear receptor co-repressor)/SMRT (silencing mediator of retinoid and thyroid receptors)] [40] or separate [CoREST (co-repressor for element-1-silencing transcription factor) (HDAC2 [41])] and are dependent on zinc (Zn^2+^) for HDAC activity [42]. Concerning the role of acetylation in cognition, it has been found that histone acetylation accompanies different forms of learning and memory, and that these acetylation changes during/following fear/extinction learning paradigms occur preferentially in learning-associated areas such as hippocampus, amygdala and prefrontal cortex [39,43] (Figure 2). Moreover, in these areas, it has been observed that histone acetylation occurs in (the promoter regions of) learning- and memory-associated genes [e.g. *Zif268* (zinc finger protein 268)/*Egr1* (early growth response 1) or *Creb1* (cAMP-response-element-binding protein)]. Thus increased histone acetylation favours gene-expression programmes necessary for memory formation. There is evidence that different learning paradigms are likely to elicit distinct epigenetic signatures, including altered histone acetylation patterns in the brain (for a review, see [39]), which seem to be region-, task- and age-specific [44]. Along these lines, research in normally extinguishing rodents has revealed that enhancing histone acetylation is a crucial molecular mechanism involved in fear extinction [41,45] (Table 1, and Figures 2 and 3). It is possible that enhancement in histone acetylation induced by fear extinction may be a co-ordinated synergistic response from both enhancement of HAT activity and reduction in HDAC activity in specific brain areas, as a recent study has revealed an increase in a HAT protein level {PCAF [p300/CBP (CREB-binding protein)-associated factor]} and reduction in HDAC2 protein levels following fear extinction learning in normally extinguishing mice [46].

**Table 1:** Studies showing that HDAC inhibitors augment exposure-based fear extinction and rescue extinction learning deficits Partial extinction: reduction of fear during the extinction training session was not to pre-conditioning levels; complete extinction: reduction of fear was to pre-conditioning levels. CaMKII, Ca^2+^/calmodulin-dependent protein kinase II; ND, not determined; NS, not stated in reference.

Compound/manipulation	HDAC selectivity	Fear reduction during extinction training	Administration of HDAC inhibitor	Histone acetylation	Behaviour	Reference
Extinguishing rodents						
Genetic knockout	HDAC2 in forebrain CaMKII neurons	Partial		ND	Increased contextual and cued extinction	[76]
Vorinostat/SAHA	Class I (HDAC1, HDAC2 and HDAC3); class II (HDAC6)*†	Complete	Immediately following fear extinction training	Increased hippocampal H3 (assessed 2 h following SAHA administration)	Increased contextual extinction	[51]
TSA (trichostatin A)	Pan class I and class II*†	Partial	Immediately before extinction training (systemic and intra-hippocampal)	ND	Increased contextual extinction	[99]
Sodium butyrate	Class I (HDAC1 and HDAC2); class II (HDAC7)‡	Complete	15 min before extinction training (systemic and intra-hippocampal)	Increased infralimbic H3K^14^ (assessed 30 min following extinction training)	Increased cued extinction and protection against spontaneous return of fear	[53]
		NS	Immediately following extinction training		Increased cued extinction	[100]
		Partial		ND	Increased cued extinction	[99]
VPA	Class I (HDAC1, HDAC2 and HDAC3)*; class I (HDAC1 and HDAC2)†	Complete and partial	2 h before extinction training	Increased prefrontal cortex H4K^5^/K^8^/K^12^/K^16^ in *BDNF* promoter I and IV (assessed 2 h following fear extinction)	Increased cued extinction	[45]
		Complete and partial	2 h before extinction training	ND	Increased cued extinction; decreased freezing to the conditioning context (renewal)	[52]
S1 mice (deficient fear extinction)						
MS-275 (entinostat)	Class I (HDAC1)*; class I (HDAC1, HDAC2 and HDAC3)†	Deficient	2 h before extinction training	ND	No fear reduction	[16]
		Complete§	Immediately following extinction training	ND	Increased cued extinction	[16]
VPA	Class I (HDAC 1, HDAC2 and HDAC3)*; class I (HDAC1 and HDAC2)	VPA induced complete extinction	2 h before extinction training	ND	Increased cued extinction	[16]
Dietary zinc restriction (ZnR)	Zn^2+^ required for HDAC activity	ZnR induced complete extinction		ND	Increased cued extinction	[56]
SPS (deficient fear extinction)						
SAHA (vorinostat)	Class I (HDAC1, HDAC2 and HDAC3); class II (HDAC6)	NS	Immediately following extinction training	Increased H3K^14^; increased H4K^5^/K^8^/K^12^/K^16^ (assessed 2 h following SAHA administration)	Increased contextual extinction	[57]

*Data obtained from assessing HDAC isoform selectivity using purified recombinant human isoforms [65].

^†^Data obtained from assessing HDAC isoform selectivity using a chemoproteomic approach [66].

^‡^Data obtained from assessing HDAC isoform selectivity in haemopoietic cell lines [101].

^§^Complete fear extinction without the administration of any pharmacological intervention was observed following ‘weak’ fear conditioning. Deficient extinction retrieval was observed revealing extinction consolidation deficits in S1 mice.

A growing corpus of data therefore indicates that pharmacological adjuncts that enhance histone acetylation can act as cognitive enhancers to augment fear extinction memory (Figure 3) in addition to other forms of extinction (e.g. [47,48]). To date, this has been assessed primarily by inhibiting HDACs as opposed to enhancing HAT activity (but see [46]). These findings show that fear reductions following extinction training can be facilitated by administration of various HDAC inhibitors, including vorinostat, entinostat, TSA (trichostatin A), sodium butyrate and VPA (valproic acid) (Table 1 and Figure 3A). Enhancing HAT activity may receive increased attention in the near future due to the development of the small-molecule HAT (CBP/p300) activator TTK21 [49]. Several studies have reported increased extinction-related brain histone acetylation and concomitant gene transcription in rodents treated with HDAC inhibitors, suggesting that enhanced gene transcription is a core mechanism supporting augmented fear extinction, in particular consolidation of extinction memory (Table 1). However, as non-histone proteins are also acetylated by HDAC inhibitors [50], it is possible that direct acetylation of non-histone proteins also contributes to enhancing fear extinction memories. Two pivotal studies have revealed that the enhancement of fear extinction, by combining extinction training with HDAC inhibitors is associated with enhancement of histone acetylation in the promoter regions of genes required for fear extinction. Specifically, promoting fear extinction with the HDAC inhibitor vorinostat [SAHA (suberoylanilide hydroxamic acid)] enhanced histone acetylation in the promoter region of NMDA receptor subunit 2B [51], a receptor (together with its downstream signalling) required for fear extinction (discussed above). The HDAC inhibitor VPA facilitates fear extinction which is associated with enhanced histone H4 acetylation in the *BDNF* (brain-derived neurotrophic factor) promoter P4 and increases in *BDNF* exon IV mRNA transcripts. BDNF is crucial for synaptic plasticity and for the maintenance of long-term memory [45] (see Figure 2C and Box 1 for details). This study provides important evidence demonstrating that histone acetylation is observed in the promoter of genes required for successful fear extinction. A significant further observation is that HDAC inhibition may also promote sustained fear inhibition and protection from fear renewal [52] or spontaneous return of fear [53] (Table 1), which would be of particular clinical importance.

### Effect of HDAC inhibitors on extinction memory deficits

The above studies have provided clear evidence that HDAC inhibition can enhance fear extinction in extinguishing rodents. Although the results of these studies are invaluable for a better understanding of the basic mechanisms of fear extinction, it is not clear whether such drugs would be beneficial in patients with PTSD, phobias or other anxiety disorder which are characterized by fear dysregulation including resistance to extinguish fear [32,54]. In this regard, findings from animal models of impaired fear extinction may have even enhanced translational value and relevance to the clinical situation. Indeed, there are a number of rodent models demonstrating impaired fear extinction (reviewed in [32]), but the present review focuses on two of these in which the utility of HDAC inhibitors to augment fear extinction training have been tested: 129S1/SvImJ (S1) mice [16,17,20,55,56] and rodents subjected to SPS (single prolonged stress) [57,58].

The use of animal models of impaired extinction has revealed an important prerequisite for the clinical use of HDAC inhibitors. Specifically, and similar to D-cycloserine (see above), rescuing fear extinction deficits via HDAC inhibition requires a significant reduction in fear during an extinction training session (i.e. a learning effect, see epigenetic priming in the ‘Safety aspects’ section below) before HDAC inhibitors (at least class I HDAC inhibitors) can be effective [16] (Table 1 and Figure 3). This has direct repercussions on the clinical treatment of anxiety disorders and suggests that multitarget approaches that induce fear reduction during extinction/CBT sessions need to be used when treating patients that do not reduce fear during CBT sessions. In this regard, the finding that the pan-HDAC inhibitor VPA was able to induce fear reduction also during an extinction training session in extinction-impaired S1 mice [16] are exciting. A summary of different strategies to rescue impaired fear extinction by combining extinction training with HDAC inhibitors is shown in Table 1. In healthy humans, it was shown recently that VPA can enhance fear extinction induced by exposure-based CBT [59,60] and can protect against fear reinstatement [60]. However, to date, no published study has investigated whether VPA is effective in augmenting exposure therapy in patients with disorders such as PTSD or phobias. An advantage of VPA over other HDAC inhibitors is that it is currently being used in psychiatric settings as a mood stabilizer [61], among other indications, and, as such, could be immediately trialled in anxiety patients. Mechanistically, VPA enhances GABAergic signalling [62,63] in addition to exhibiting predominately class I HDAC inhibition [64–66]. It is attractive to speculate that (i) enhancement in GABAergic activity may be involved in initiating the extinction training effect in S1 mice as specific GABAergic activity contributes to the molecular mechanisms underlying fear extinction acquisition (see, for example, [67–70]), and that (ii) enhancement of histone acetylation, via VPA's HDAC-inhibitory properties corrected and prolonged impaired fear extinction consolidation.

Box 1 **BDNF is a critical neurotrophic factor under extinction-induced epigenetic regulation**

Consolidation of fear extinction memories requires synaptic plasticity within discrete regions of an amygdala–medial prefrontal cortex–hippocampal circuit mediating fear extinction [91]. Of known neurotrophic factors, BDNF is emerging as an important regulator of extinction-induced plasticity [92]. Pre-clinical studies have shown that rodents exhibiting either reduced global or basal amygdala levels of BDNF fail to extinguish fear [78,93], and, conversely, boosting BDNF in extinction-relevant brain regions (medial prefrontal cortex or hippocampus) can facilitate fear extinction [93,94] and can promote the formation of enduring fear inhibitory memories [104]. Further underscoring the importance of BDNF signalling in extinction mechanisms are the findings that humans and mice carrying the Met allele of the BDNF V66M polymorphism show impaired fear extinction [95]. It is known that HDAC inhibitors, including VPA, alter the transcription of a small proportion of existing genes (approximately 9%) including *BDNF* [82,96]. In this regard, it is possible that enhanced transcription of *BDNF* may contribute to the rescue of impaired fear extinction induced by HDAC inhibitors including VPA (see the main text and Table 1). Indeed, transcription of *BDNF* is under epigenetic regulation, as enhanced histone acetylation is observed in the promoter region of *BDNF* exon IV following successful fear extinction, augmented by the pan-HDAC inhibitor VPA [45]. Collectively, these studies indicate that BDNF is an important downstream target of HDAC inhibitor-induced facilitation of extinction (Figure 2).

Moreover, research has revealed that chronic (oral) fluoxetine (an SSRI) treatment increases BDNF expression in extinction-relevant brain regions including the medial prefrontal cortex and hippocampus [97]. Indeed, combining chronic fluoxetine administration with fear extinction rescues impaired fear extinction in S1 mice [79] and promotes sustained basal amygdala- and BDNF-dependent fear inhibition in normally extinguishing mice [78]. It remains to be assessed whether chronic fluoxetine and other treatments enhance histone acetylation in the promoter region of BDNF also in animal models with impaired extinction.

An important point is to explain how is it possible that systemically administered HDAC inhibitors (and chronic fluoxetine) combined with extinction trials seem to exert their effects preferentially in brain areas involved in learning and memory. One answer might lie in epigenetic priming, (reviewed in [39]), where successful CBT/extinction training in rodents with intact fear extinction can trigger histone acetylation changes in key extinction-related brain regions (see the main text), resulting in ‘relaxed chromatin’ (Figure 2C) around genes required for the consolidation of this extinction memory (including *BDNF*). HDAC inhibitors, applied at the time of CBT/extinction training, can facilitate transcription of these genes as they have been ‘primed’ through activity related to successful CBT/extinction training. S1 and SPS rodents may exhibit deficient fear extinction due to lack or insufficient ‘epigenetic’ priming in relevant areas (see the main text). Thus HDAC inhibitors applied following successful fear reduction may serve to compensate for the insufficient priming and subsequent acetylation to promote long-term fear inhibitory memories.

It is not yet clear whether extinction-impaired animals show evidence of acetylation dysregulation, e.g. by disruption of HAT/HDAC balance, as reported from other forms of cognitive dysfunction [71,72]. Hence it is important to explore chromatin status and gene expression of well-established extinction genes in these animal models. Indeed, in addition to transcriptional dysfunction in S1 mice, indicated by insufficient expression of the memory-related gene *Zif268* in extinction relevant areas (infralimbic cortex, intercalated cell mass nucleus, basal and centrolateral amygdala) [17,56], preliminary data from our laboratory now indicate that histone hypo-acetylation in the amygdala also accompanies the extinction memory impairment in S1 mice, potentially providing a mechanism contributing to blunted gene transcription. Both amygdala acetylation and extinction deficits were restored by HDAC inhibitor treatment. Moreover, rescuing deficient fear extinction with HDAC inhibitors produced a context-independent fear extinction memory which was enduring as protection against spontaneous fear recovery and fear renewal in a novel environment was observed (N. Whittle, M. Haus-child, S.B. Sartori, V. Maurer and N. Singewald, unpublished work). These results extend findings in rodents with intact fear extinction revealing that enhancing histone acetylation, via increasing HAT activity, can enhance fear extinction that is context-independent [46]. Collectively, these results indicating that HDAC inhibition promotes context-independent and enduring fear extinction memories in pre-clinical models support evidence that HDAC inhibitors may be used as cognitive enhancers to rescue fear extinction deficits.

## Future development of HDAC inhibitors for anxiety and trauma therapy

### Which HDAC isoform(s) should be targeted?

The development of isoform-specific HDAC inhibitors to more precisely modulate epigenetic mechanisms has proven challenging [73]. However, some advances have been made, such as class I-specific inhibitors with nanomolar potency, as well as some rare examples of single isoform specificity (see below). An important aim currently in the fear and anxiety field is the identification of the HDAC isoform(s) contributing to or mediating enhanced fear extinction. As can be seen in Table 1, current commercially available HDAC inhibitors used so far exhibit little isoform-specificity (i.e. pan-HDAC inhibitors), with the exception of MS-275 or RGFP966. Complicating the issue, there is evidence that the specificity of HDAC inhibitors might be directed not towards HDAC isoforms, but rather towards HDAC complexes such as CoREST or NuRD [66]. Moreover, the clinical use of pan-HDAC inhibitors could be problematic as recent targeted gene-deletion studies have provided evidence that individual HDAC isoforms serve very distinct functions within the central nervous system [35,74]. The currently used HDAC inhibitors in learning and memory exhibit the greatest selectivity to class I HDACs (encompassing HDAC1, HDAC2, HDAC3 and HDAC8) [75]. This suggests that targeted development of compounds that inhibit specific class I HDAC isoforms be pursued. Reinforcing this notion is the finding that genetic ablation of HDAC2, which binds to synaptic plasticity-related genes, including *ZIF268*, *CREB1*, c-*FOS*, *BDNF*, *CBP*, *NRN1* (neuritin 1) and NMDA receptor subunit genes, enhances LTP (long-term potentiation) [41] and fear extinction learning in normally extinguishing mice [76]. These data reveal HDAC2 as a prime HDAC isoform for which targeted pharmaceutical compounds could be developed to augment fear extinction. However, knowledge of the involvement of additional class I HDAC isoforms is warranted given that specific class I HDAC isoforms have been implicated in other forms of learning and memory processes.

To date, the role of HDAC1 in exposure-based fear extinction is not clear. An interesting study has shown an enhancement or impairment of an extinction-like effect when HDAC1 in the hippocampus was overexpressed or genetically deleted respectively [77]. This finding is somehow contradicting findings with pan-HDAC inhibitors (see above). Since the researchers used a paradigm which consisted of daily short (3 min) fear exposure (extinction) sessions, there are, however, alternative possibilities of interpreting these data. In brief, following fear expression, a wave of gene transcription, akin to that following CBT-based fear extinction (see the ‘Quantifying fear and fear extinction in pre-clinical animal models’ section above), is required to (re)consolidate the recently retrieved fear memory, which has been transferred into a labile short-term memory, into a stable long-term memory. It could be argued that, e.g., reducing hippocampal HDAC1 levels (which resulted in increased acetylation and c-*FOS* gene transcription) promoted fear memory reconsolidation rather than affecting (impairing) extinction learning directly. Interestingly, genetic ablation of HDAC1 does not alter fear learning [41]. As there are examples of pharmaceutical treatments that facilitate CBT-based fear extinction, but not fear conditioning (including dietary zinc restriction [56], chronic fluoxetine administration [78,79] and enhancing endocannabinoid [20] or metabotropic glutamate receptor 7 [16,80,81] signalling), and, furthermore, individual HDAC isoforms can regulate specific forms of learning, while leaving others unaffected [74], it remains to be tested whether enhancing gene transcription, via HDAC1 inhibition, can enhance fear extinction memories induced by CBT-based (massed) fear extinction. Clarification is also warranted as genetic knockdown of hippocampal HDAC1 has been shown to enhance histone acetylation in *BDNF* promoter exon IV and to increase expression of *BDNF* exon IV mRNA transcripts [82], suggesting that interaction of this HDAC isoform with the regulation of a gene is required for consolidation of extinction memories (Figure 2 and Box 1). HDAC3, similar to HDAC2, has been considered as a molecular brakepad for long-term memory formation [41,83]. Genetic ablation or pharmacological (by RGFP966) inhibition of HDAC3 enhances cognitive performance in a spatial learning and memory task [84] and augments extinction of conditioned cocaine place preference [48]. These studies suggest that HDAC3 inhibition may also augment fear extinction, which has yet to be assessed. Finally, HDAC8 can negatively regulate CREB-dependent gene expression [85], a transcription pathway critical for the transcription of genes required for consolidation of extinction memories [86]. This study may suggest that HDAC8 inhibition be a candidate target for facilitation of fear extinction.

The involvement of other HDAC classes in extinction has received little attention to date. However, emerging evidence suggests that, in addition to class I HDACs (described above), specific class IIa HDAC isoforms (HDAC4, HDAC5, HDAC7 and HDAC9) may also have the potential to modulate CBT-based fear extinction. Selective brain-specific loss of HDAC4 [87] or HDAC5 [88], which interacts with HDAC3-containing complexes to exert HDAC activity has recently been shown to impair fear learning and spatial learning and memory, which is associated with impaired long-term synaptic plasticity. To date, there is no published study investigating the influence of HDAC7 or HDAC9 on cognitive processes. Collectively, the above studies may suggest that, opposite to class I HDAC isoforms, increasing the expression of class IIa isoforms may facilitate specific forms of learning and possibly also CBT-based fear extinction; however, this remains to be tested. Moreover, these results at present would suggest that future development of HDAC inhibitors should restrict specificity to class I HDAC isoforms.

### Safety aspects

Despite early reports of possible neurotoxic effects of some HDAC inhibitors [89], most of these drugs are remarkably well tolerated, in both humans and other animals, where they are mainly administered chronically as anti-cancer agents. Reported side effects include diarrhoea, myelosuppression and cardiac QT persistence [90]. As HDAC inhibitors are suggested to be predominantly acutely administered to augment CBT therapy, it may be expected that even these side effects are somewhat mitigated. In principle, systemically applied HDAC inhibitors act globally in targeted and non-targeted tissues, opening the possibility of widespread off-target effects. Interestingly, however, for example in learning and memory, there seems to be (at least to some extent) gene and brain area specificity of acetylation changes. ‘Epigenetic priming’ was postulated as a possible explanation for these observations, stating that HDAC inhibitors have more pronounced effects in those particular genes and brain areas, which, by learning, have already been primed via associated neural activity-induced chromatin remodelling [39]. Thus, according to this hypothesis, HDAC inhibitors would reinforce already occurring (but in some cases insufficient) gene expression activity, whereas its effect in other brain areas or peripheral tissues with low baseline activity would be minimal. However, future studies will need to carefully clarify in particular the long-term consequences of HDAC inhibitor treatment in targeted and non-targeted tissues.

## Conclusions

A growing corpus of data in healthy humans and rodents shows that inhibition of HDACs can act as cognitive enhancers to facilitate extinction-based therapeutic approaches and render these effects particularly enduring. Novel evidence that HDAC inhibitors can rescue extinction deficits in extinction-impaired rodents further underscores the potential clinical utility of this approach also for exposure therapy-resistant patients. However, it is clear that further research, particularly also in a pathological context (i.e. impaired extinction) is needed to identify the specific contributions of individual HDAC isoforms, or their combinations, as well as the protein complexes in which HDACs associate in regulating extinction-related cognitive performance. Additional important aims are in gaining greater information on pharmacokinetic parameters and blood–brain penetrance of HDAC inhibitors. With this information, it will be possible to rationally exclude non-specific action of HDAC isoforms not involved in augmenting fear extinction and thereby reduce potential adverse effects of this approach. Moreover, as HDAC inhibitors can enhance fear learning and fear reconsolidation [35,52], the clinical timing of HDAC inhibitor administration must be carefully controlled and, similarly to D-cycloserine [13], applied only following successful fear reduction induced during exposure-based therapy. Notwithstanding, given the striking translational value of findings in this field [8], the existing pre-clinical evidence described in the present review clearly provides the impetus for follow-up studies to be conducted across different fear- and trauma-related disorders to evaluate whether and how inhibiting the activity of individual or combined HDACs in the brain can augment therapeutic effects induced by CBT.

## Note added in proof (received 30 January 2014)

While the present article was in proof, an important study was published demonstrating the role of the HDAC2 isoform in promoting persistent fear extinction in rodents [105].
